# Supplementary material for: Dynamic Changes in the Distribution of Hydrocodone and Oxycodone in Florida from 2006 to 2021
Source: Pharmacy (Basel). 2024 Jun 28;12(4):102. doi: 10.3390/pharmacy12040102 (PMC11270220; doi:10.3390/pharmacy12040102)
Supplement: Supplementary file 1 [file pharmacy-12-00102-s001.zip › pharmacy-3038795-supplementary.pdf]

Supplementary Materials: Dynamic Changes in Distribution of Hydrocodone and Oxycodone in Florida from 2006 to 2014

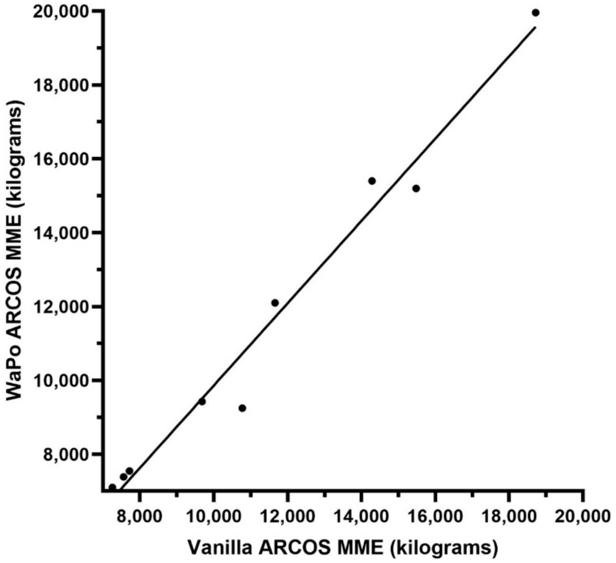

Figure S1. Correlation between Washington Post ARCOS data and Vanilla (traditional) ARCOS data. Measured by comparing the MMEs in kilograms for the year 2010 calculated by each dataset. R^2=0.98.

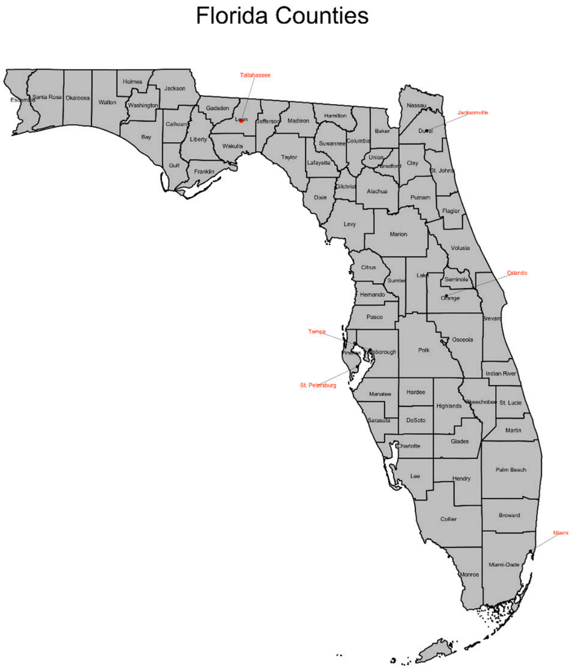

Figure S2. Map of Florida's counties.
